# Introducing a Regulatory Sandbox Into the Indonesian Health System Using e-Malaria as a Use Case: Participatory Action Study

**DOI:** 10.2196/47706

**Published:** 2023-12-05

**Authors:** Anis Fuad, Agi Tiara, Rizqiani Amalia Kusumasari, Rimawati Rimawati, E Elsa Herdiana Murhandarwati

**Affiliations:** 1 Department of Biostatistics, Epidemiology, and Population Health Faculty of Medicine, Public Health, and Nursing Universitas Gadjah Mada Yogyakarta Indonesia; 2 Center for Tropical Medicine Faculty of Medicine, Public Health, and Nursing Universitas Gadjah Mada Yogyakarta Indonesia; 3 Department of Parasitology Faculty of Medicine, Public Health, and Nursing Universitas Gadjah Mada Yogyakarta Indonesia; 4 Faculty of Law Universitas Gadjah Mada Yogyakarta Indonesia

**Keywords:** regulatory sandbox, digital health, disruptive technologies, e-malaria, participatory action research, Indonesia

## Abstract

**Background:**

Regulatory sandboxes offer an alternative solution to address regulatory challenges in adopting disruptive technologies. Although regulatory sandboxes have been widely implemented in the financial sector across more than 50 countries, their application to the health sector remains limited.

**Objective:**

This study aims to explore stakeholders’ perspectives on introducing a regulatory sandbox into the Indonesian health system using e-malaria as a use case.

**Methods:**

Using a participatory action research approach, this study conducted qualitative research, including desk reviews, focus group discussions, and in-depth interviews with stakeholders. This study sought to understand stakeholders’ concerns and interests regarding the regulatory sandbox and to collaboratively develop a regulatory sandbox model to support the malaria program.

**Results:**

The study revealed that most stakeholders had limited awareness of the regulatory sandbox concept. Concerns have been raised regarding the time required to establish regulations, knowledge gaps among stakeholders, data protection issues, and limited digital infrastructure in malaria endemic areas. Existing regulations have been found to be inadequate to accommodate disruptive healthtech for malaria. Nevertheless, through a collaborative process, stakeholders successfully developed a regulatory sandbox model specifically for e-malaria, with the crucial support of the Ministry of Health.

**Conclusions:**

The regulatory sandbox holds the potential for adoption in the Indonesian health system to address the limited legal framework and to facilitate the rapid and safe adoption of disruptive healthtech in support of the malaria elimination program. Through stakeholder involvement, guidelines for implementing the regulatory sandbox were developed and innovators were successfully invited to participate in the first-ever trial of a health regulatory sandbox for e-malaria in Indonesia. Future studies should provide further insights into the challenges encountered during the e-malaria regulatory sandbox pilot study, offering a detailed account of the implementation process.

## Introduction

### Background

Regulatory sandboxes have been widely implemented in the financial sector to facilitate adoption of disruptive technologies. More than 50 countries, including Indonesia, have adopted this framework [1]. The COVID-19 pandemic has further underscored the importance of regulatory sandboxes, as evidenced by the use of emergency authorization for COVID-19 vaccines [2,3]. Although regulatory sandboxes have been used in a number of health sectors in high-income countries [4,5], their adoption remains very limited in low- and middle-income countries, including Indonesia.

Regulatory sandboxes in the health sector strike a balance between disruptive innovation and public health [2]. They created a safe testing ground in which new regulatory processes and technologies can be evaluated in real-world settings. By providing a real-world testing environment, regulatory sandboxes can provide valuable evidence on the effectiveness and feasibility of digital health interventions. This allows for the cautious relaxation of certain regulations to foster innovation, while ensuring that new technologies undergo validation before widespread implementation. Ultimately, regulatory sandboxes enable the scale-up of proven digital healthtech [3,4].

Although a framework exists for policymakers to implement a regulatory sandbox [1], there is currently no specific framework tailored to health policymakers. However, the health sector actively advocates for the implementation of digital healthtech to enhance health systems. The World Health Organization (WHO) has been at the forefront of publishing guidelines on digital health interventions, including the recent release of digital adaptation kits for specific health programs and diseases, such as WHO smart guidelines for antenatal care, family planning, and HIV [6,7]. Furthermore, an increasing number of innovative health solutions have been developed for the treatment of various diseases.

Indonesia is a thriving digital ecosystem hub in Southeast Asia, with a robust millennial population and numerous unicorn start-ups. The country has witnessed remarkable growth in the digital technology sector [8]. Notably, during the pandemic, the Indonesian Ministry of Health (MoH) collaborated with 17 start-ups to deliver teleconsultation services to COVID-19 self-isolated patients. According to the Indonesian Telemedicine Alliance, telemedicine providers have conducted a staggering 17.9 million teleconsultations during the pandemic [9]. Unfortunately, the regulatory framework for these emerging businesses remains inadequately defined, as the MoH currently lacks specific regulations governing health care start-ups. Instead, these entities primarily fall under the regulatory purview of the Ministry of Communication and Informatics as electronic system providers. This regulatory discrepancy raises concerns regarding oversight of safety and quality control for health care start-ups operating within the country.

### Objectives

This study aimed to explore stakeholders’ perspectives on this issue by introducing a regulatory sandbox approach. Unlike studies that have primarily focused on well-known health issues, our investigation focused specifically on malaria, a persistent public health issue in Indonesia, particularly in the eastern region. Although there has been significant progress in reducing malaria cases over the past decade [10], advancements have stagnated since 2014 because of limited resources, funding, regulations, and challenging geographic conditions. Embracing digital technology is crucial for effective malaria elimination, aligned with the WHO Global Technical Strategy for Malaria 2016-2030 [11-13]. However, the lack of adaptive regulation of disruptive technologies poses a potential threat to the effective elimination of malaria.

The regulatory sandbox concept is relatively new to the health sector, requiring a thorough understanding and compelling use cases that present clear opportunities for involving digital innovators to join the safe testing experiment [4]. In this study, we introduced e-malaria, encompassing various digital innovations that can contribute to malaria programs, including prevention, control, diagnostics, treatment, and surveillance. Recognizing the importance of stakeholder engagement in health policymaking and regulation [14], this study aimed to explore stakeholders’ comprehension and expectations regarding the introduction of a regulatory sandbox to facilitate the adoption of disruptive technologies in the context of e-malaria.

This study is expected to contribute to the understanding of regulatory sandboxes in the global health context. Several publications have mentioned pilot projects, their limited sustainability, and their use as an alternative method to healthtech assessment [4,15]. By exploring stakeholders’ perspectives and involvement in introducing a regulatory sandbox into the Indonesian health system, specifically using e-malaria as a use case, this study aimed to provide valuable insights and contribute to the broader discourse on regulatory sandboxes in health care.

## Methods

### Study Setting

This study was conducted in the context of the Indonesian health system, which faces challenges related to the limited understanding of a regulatory sandbox as an example of a progressive law approach. In the Indonesian health system, there are an increasing number of healthtech companies operating without specific regulations and supervision from the MoH. Instead, these companies are primarily regulated by the Ministry of Communication and Informatics regarding the registration of electronic service providers.

To gain a comprehensive understanding of stakeholders’ perspectives on regulatory sandboxes within the Indonesian health system, we used a participatory action research approach with qualitative data collection methodology. This approach, which has been practiced elsewhere, allows for active engagement and buy-in of the stakeholders [16].

Data were collected through 7 focus group discussions (FGDs) and 2 in-depth interviews, all of which were conducted online because of the constraints imposed by the COVID-19 pandemic. This web-based format allowed remote participation and ensured the safety and convenience of participants. Data collection was conducted between April 2020 and December 2021, allowing a comprehensive exploration of stakeholder perspectives over an extended period.

By conducting this study within the specific context of the Indonesian health system, where the understanding of regulatory sandboxes is limited, we aimed to provide valuable insights that can inform policy development and decision-making processes related to health sector regulation and supervision.

### Study Design

To achieve the objective of this study, we used a participatory action research approach using a qualitative methodology. Participatory action research is grounded in theoretical frameworks and previous studies that emphasize the collaboration, engagement, and active participation of stakeholders throughout the research process. It aims to generate knowledge and bring about social change by involving stakeholders at every stage of the study [17-19].

**Figure 1:**
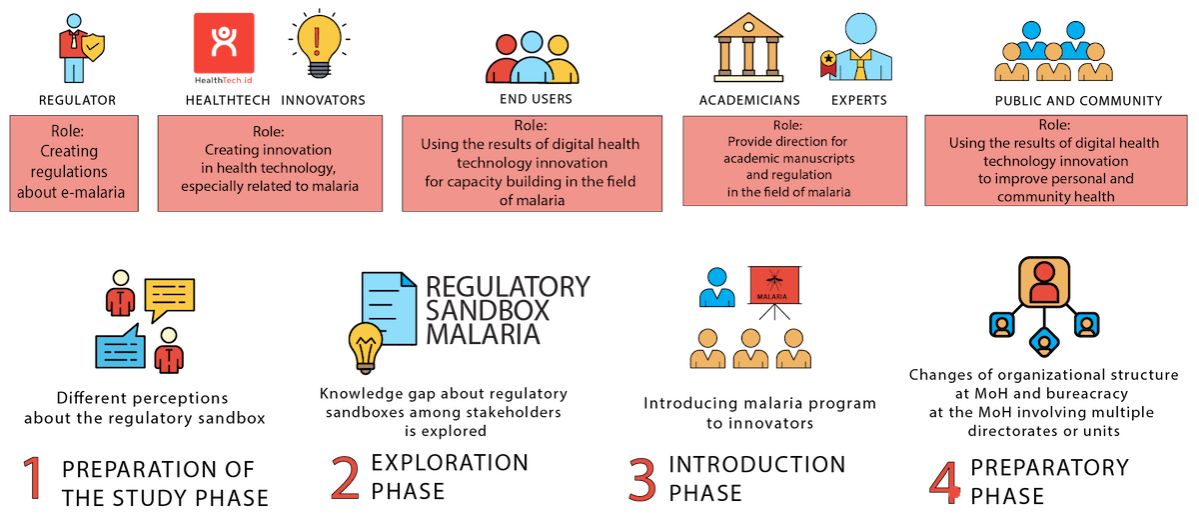
Stakeholders’ roles and stages of the study. MoH: Ministry of Health.

This study consisted of a series of stages, including a preliminary study, exploration of stakeholders’ understanding, introduction of the regulatory sandbox concept, and completion of the preparatory phase of regulatory sandbox trials in e-malaria (Figure 1). The involvement and contribution of stakeholders in these stages ensures that their perspectives and experiences are central to the research process. This approach also demonstrates the value of engaging stakeholders in research processes [19].

The identification of participants and stakeholders was a crucial aspect of the study design. Various stakeholders, including regulators from the MoH, innovators, academics, practitioners, and implementers or users were involved. These stakeholders were selected based on their expertise, involvement in the malaria program, familiarity with disruptive healthtech, and regulatory expertise. Their diverse perspectives and roles ensured a comprehensive understanding of the subject matter and enriched the quality of the findings.

### Data Collection

The data collection process involved in-depth interviews and FGDs to gather comprehensive insights into stakeholders’ perspectives regarding the introduction of a regulatory sandbox for e-malaria. The collected data were systematically coded and organized according to the topics discussed to facilitate analysis and interpretation.

The main points and concerns of stakeholders regarding the implementation of the regulatory sandbox were identified and described. These FGDs and seminars were virtually held. In addition, we held a talk show at the launch of the e-malaria regulatory sandbox guidelines, which was attended by stakeholders and the media.

All sessions, including FGDs, interviews, and talk shows, were recorded and transcribed to ensure accuracy during the data analysis phase. These transcriptions serve as valuable resources for capturing the nuances and details of stakeholders’ perspectives of this new approach. Guidelines for FGDs and interviews were developed to ensure consistency and facilitate gathering of perspectives from diverse groups of stakeholders. These guidelines served as a framework for the data collection process, ensuring that key topics were explored and enabling a thematic analysis of the collected data.

To provide a comprehensive overview of the study, Table 1 outlines the role of stakeholders at various stages of the research process, whereas Tables 2 and 3 present the themes and participants involved in FGDs, virtual seminars, and talk shows. These tables offer a clear snapshot of the data collection methods and participants involved in each phase of the study, aiding in the organization and interpretation of the findings.

In addition to the primary data collection methods, a desk review of the relevant Indonesian policies and regulations related to digital health and malaria was conducted. This review provides a contextual understanding of the existing landscape of various disruptive healthtech, especially those in malaria, and an analysis of stakeholders’ perspectives.

For in-depth interviews and FGDs, the interviews and FGD instruments are available in Multimedia Appendix 1, with detailed information on the questions and prompts used to elicit stakeholder responses. The number of FGD respondents is presented in Table 2, demonstrating the size and diversity of the participants involved in FGDs. In cases where opinions were not homogenous within an FGD, in-depth interviews were conducted to capture a more nuanced understanding of stakeholders’ perspectives. To ensure ethical considerations, all the informants who participated in the FGDs and interviews received compensation for transportation costs or internet quotas, acknowledging their time and contribution to the research process.

**Table 1:** Roles of stakeholders during the study stages.

	Preliminary (April-July 2020)	Exploration (August-December 2020)	Introduction (January-June 2021)	Preparatory (June-December 2021)
Stakeholders^a^	Regulators, innovators, and users	All	All	All
Roles of stakeholders	Signing the memorandum of agreement between the Faculty of Medicine, Public Health, and Nursing Universitas Gadjah Mada with the Directorate of vector-borne diseases and zoonosis of MoH^b^	Participants and resource persons of a kickoff meeting and virtual seminar	Participants and resource persons of FGDs^c^ and the virtual seminar	Participants and resource persons of FGDs and the virtual seminar
Challenges and findings	Different perceptions about the regulatory sandbox	Knowledge gap about the regulatory sandbox	Limited understanding by healthtech innovators regarding the malaria program Majority of innovators have experience as telehealth service providers	Changes of organizational structure at the MoH Complexities of coordination between units at the MoH
Result	Proposal for funding	Issues, challenges, and opportunities for implementing the regulatory sandbox have been explored	Identified healthtech for malaria Consensus on cluster of disruptive technologies Launching the regulatory sandbox guideline	Decree from MoH on reviewer Web-based application for sandbox registration Socialization and call for participation

^a^Categories of stakeholders: regulators, innovators or healthtech companies, academicians or experts, users, and public or community.

^b^MoH: Ministry of Health.

^c^FGD: focus group discussion.

**Table 2:** Focus group discussions (FGDs) to develop the concept and model of an e-malaria regulatory sandbox.

FGD	Title and aims	Respondents; n
FGD 1	Overseeing regulation and governance for malaria (stakeholders) Areas explored: Acceptance of the digital technology and challenges in malaria: legal-ethics, sociocultural, and financial issues of e-malaria from different perspectives Prospect of implementation of e-malaria in national scope	Provincial health services, regional public and private hospitals, and other health facilities that provide malaria check-up services, and related parties; 19
FGD 2	Overseeing regulations and governance of e-malaria (health officers) Aims: Evaluating the existing regulations and governance in malaria and prospect to implement e-malaria Identifying the prospect of e-malaria in Indonesia along with possibilities of business prospect Understanding the various points of views and possibilities and challenges regarding ethico-legal, sociocultural, and financial issues of e-malaria along with future anticipation	Health officers and a malaria program supervisor from various provinces in Indonesia; 22
FGD 3	Overseeing regulations and governance of e-malaria (MoH^a^ as regulator) Aims: Identifying the significance of and strategic issues regarding changes and development in healthtech dynamics, especially e-malaria technology; ethico-legal and socioeconomic appropriateness; and systems supporting implementation at the national level Understanding the challenges and probabilities of malaria e-diagnostic development regarding their the ethico-legal, sociocultural, and financial aspects along with future anticipation	Indonesian MoH as the regulator, Malaria Expert Committee, and other malaria experts from various research committees; 19
FGD 4	Overseeing regulations and governance of e-malaria (start-up) Focuses on standard technology Aims: Identifying the significance of and strategic issues regarding changes and development in healthtech dynamics, especially e-malaria technology; ethico-legal and socioeconomic appropriateness; and systems supporting implementation at the national level Understanding the challenges and probabilities of malaria e-diagnostic development regarding their ethico-legal, sociocultural, and financial aspects along with future anticipation Understanding digital health start-ups’ point of view toward future regulatory sandbox models and current regulations	Digital health start-ups, digital initiatives, and regulators, such as MoH; 22
FGD 5	Compiling documents regarding future implementation of governance concept on a limited scale Aims: Adjusting governance model according to the on-site users Understanding experts’ point of view toward existing telemedicine regulations Compiling inputs about the future regulative measurement toward e-malaria and the regulatory sandbox	Health law and technology law experts, health experts from various disciplines, IT experts, and regulators; 11
FGD 6	Compiling documents on improving the governance concept Aims: Improving the adjusted governance model for the on-site users’ needs according to the previous FGD Improving the governance model supported by regulators from previous FGD Receiving substantive input about the regulatory sandbox model for e-malaria from regulators and experts	Health law and technology law experts, health experts from various disciplines, IT experts, and regulators; 11
FGD 7	Drafting the governance concept document and early drafts of the academic script Aims: Compiling the academic draft as a requirement to create a new regulation Drafting the governance concept document Policy brief drafting regarding the regulatory sandbox and its pilot Adjusting the current academic draft for hearing preparation with regulators	Health law and technology law experts, health experts from various disciplines, IT experts, and regulators; 16

^a^MoH: Ministry of Health.

**Table 3:** Virtual seminar and talk show to introduce the potential of a regulatory sandbox approach in the malaria program.

Virtual seminar and talk show	Title	Participants; n
Virtual seminar 1	Telemedicine and artificial intelligence in a malaria elimination program in Indonesia	Academicians, researchers, students, start-up companies, and government employees; >180
Virtual seminar 2	Telemedicine legal studies: the potential of a regulatory sandbox approach	Academicians, researchers, students, start-up companies, and government employees; >200 Resource persons: OJK^a^ Indonesia; the Ministry of Health, Singapore; and a Singaporean start-up
Virtual seminar 3	Welcoming the regulatory sandbox in the health sector and the e-malaria regulatory sandbox handbook	Academicians, researchers, students, start-up companies, and government employees; >100
Talk show	Potential and challenges of healthtech for nonprofit and humanitarian programs	Academicians, researchers, students, start-up companies, and government employees; 30

^a^Otoritas Jasa Keuangan*,* also known as the Indonesian Financial Services Authority.

### Data Analysis

Thematic analysis was used to analyze the collected qualitative data. The research team members collaborated to develop the research protocol and identify thematic issues. A total of 2 researchers (AT and RAK) took the lead in developing probes and interview questions for in-depth interviews and FGDs, respectively. To verify the results, team members evaluated and discussed the theme analysis. This iterative procedure provides many perspectives and interpretations that improve the reliability of findings. Thematic analysis began in Indonesian, the data collection language. The analysis was translated into English to publish the findings. For further inquiries or access to qualitative data, interested parties may contact the research team directly.

### Ethical Considerations

This study was approved by the Ethics Committee of the Faculty of Medicine, Public Health, and Nursing at Universitas Gadjah Mada (KE/FK/0644/EC/2020). This ensured that the research was conducted in accordance with established ethical standards, thus safeguarding the rights and risks of the participants.

## Results

### Overview

This section presents the findings that explain the 4 stages of the study: the preliminary, exploration, introduction, and preparatory phases of the regulatory sandbox for e-malaria. To provide a clear and comprehensive description, 4 tables, 2 figures, and 4 textboxes are included to illustrate the perspectives of the stakeholders and their involvement in this study.

The findings discussed in this section include the roles of stakeholders, the detailed activities of FGDs and virtual seminars, and tables that provide an in-depth analysis of the issues and concerns raised by stakeholders in each phase (Tables 1-4 and Textboxes 1-3). In addition, a selection of quotes from the respondents is presented to reinforce the themes summarized in the tables.

**Table 4:** Perspectives of stakeholders on introducing regulatory sandboxes in the health sector.

Stakeholders	Pros	Cons
Regulators	The current regulations do not adequately accommodate the development of healthtech in the field, whereas the technology is rapidly developing and needs regulations to support the innovations.	Financing for the malaria program should be discussed with the regional government—although currently the financial burden is borne by central and district state budgets, it is still necessary to advocate for the implementation budget to regional leaders. Each region has its own regulations, as new regulations will cause new confusion in the implementation. There is a need for clarity on regulations and stakeholders to encourage acceptance of regulatory sandbox regulations among all stakeholders involved.
Innovators	The regulatory sandbox will provide a sense of security (to innovation users) and will indirectly assist in validating applications.	From the perspective of the Healthtech Association, it takes a long time from the regulatory sandbox idea acceptance to become a regulation.
Academicians	Downstreaming of superior disruptive innovation products can be adopted quickly.	From a legal perspective, a new regulation will need model testing, public testing, and jurisdictional review, which will take a long time.
Public	There are many health businesses that use information technology. Licensing and testing arrangements are crucial to ensure comprehensive protection in all aspects.	Health data are personal data that should be protected by the government. This approach requires a mindset that relies on consumer protection.
Users	With digital health innovations in the malaria field, this can save budget and time and make it easier for analysts in the field.	Infrastructure barriers for areas with limited or poor internet access.

**Summary of the exploration stage.**

**Technical situation in the field**

- Ability to identify the malaria problems in the diagnosis, quality control and quality assurance because of limited human resources, transportation and orgeographic, and funding
- Ability to present solutions, such as telehealth, IT use, information system, and the role of regulations in hastening the adoption of innovations
- Ability to identify required clusters for e-malaria
- IT literacy: WhatsApp group as the consultation room, telemedicine, and malaria program
- Support from providers or start-up and associations
- Internet connection relatively available, albeit with varying distribution or speed
- Support from stakeholders, users, providers, local governments, and partners

**Law and social issues**

- Legal guarantee issues for users, providers, public, and program
- Data security and data privacy issues
- Needs for progressive law
- Socio-legal aspect

**Challenges and opportunities**

- Positive law regulation regime
- Used for the first time in the Ministry of Health
- Malaria as a humanitarian problem
- Laws as a means for humanity to prosper
- Successful adoption of regulatory sandboxes in Indonesia’s financial sector

**Summary of the stakeholders’ responses during the introduction section stage of the e-malaria regulatory sandbox.**

**Expectation**

- Innovation and digital transformation are needed to help the malaria program.
- Digitalization will help malaria in endemic areas, particularly in the eastern part of Indonesia.
- Regulatory sandboxes could be a new initiative to develop new ecosystems among government and private communities.

**Technology-related issues**

- Advanced healthtech introduced by start-ups need to be anticipated. The higher the technology, the higher the bandwidth.
- Bandwidth could be a particular issue, as coincidentally the malaria endemic areas are located in limited-infrastructure areas.
- New healthtech (digitalization) should be integrated into the established system, National Health Insurance Agency, and other health data.
- Regulatory sandboxes are expected to develop a new ecosystem in the public and private sectors and society, leading to digital transformation in the health sector.

**Legal-related issues**

- There are concerns related to patient, user, and provider protection.
- Issues regarding digital literacy and the legal basis to prevent data leakage.
- Legal certainty during data breaches.
- Foreign-funded start-ups have potential data ownership issues.
- The Ministry of Informatics and Communication is involved in digital health regulation.
- Simplification of regulation is needed. Regulation of medical records should be adapted to the digital ecosystem.
- Academic drafts should accommodate all aspects.

**Participants’ responses toward the preparation phase of the regulatory sandbox for e-malaria.**

**Law-related issues**

- Will this piloting become a national program?
- Regulation should be general on the national level, while adoption should be led by regional governments in accordance with their own characteristics, for example, regional autonomy.
- Data security should be the responsibility of the Ministry of Health.
- The law should be a reference but should not be settled without considering its urgency and benefits.
- It is necessary to think about the process of the relationship between the law and the authorizing body.

**Technologies, funding, and other issues**

- Budgeting and technical instructions for implementation are required.
- For malaria programs at the provincial level, there is already a government regulation, but if the regulatory sandbox is implemented, a user might need technical instructions from the district health office.
- Internet users in Indonesia comprise >70% of the population and can be expected to increase in the future.
- Big data will be useful when managed correctly.
- There is a need to map technology readiness and digital literacy.
- Developed clusters might need to pay attention to the available infrastructure.

**Feasibility**

- Legal acts are needed when piloting and adopting regulatory sandboxes into a national program.
- Regulations with general characteristics are preferable to detailed ones.
- Licensing can be strict, but regulations require a light touch.
- The balance between regulation safety and agility must be found.
- Across borders and across sectors, regulations are increasingly easy to make but difficult to implement.
- There are needs for risk mitigation and privacy impact assessment.
- There are needs to assess acceptability, budget waste, and exit strategies after the research is completed.

The perspectives of stakeholders and their involvement in the development of the regulatory sandbox are reflected in the stages of this study as well as in activities such as FGDs, virtual seminars, and talk shows. Several findings were identified through these activities, including the challenges and limitations faced by stakeholders as well as positive aspects that could potentially support the development of a regulatory sandbox. The exploration, introduction, and preparation of the e-malaria regulatory sandbox trial were informed by a sequence of FGDs that covered diverse themes, objectives, and participants (Table 2).

In addition to FGDs aimed at exploring stakeholder perspectives, this study also conducted virtual seminars open to the public. The seminars aimed at knowledge exchange and featured speakers from the MoH of Singapore and a Singaporean start-up. The speakers shared valuable insights and experiences regarding the implementation of the regulatory sandbox, specifically discussing the Licensing Experimentation and Adaptation Programme for telemedicine in Singapore, which has been in operation since 2018. Local speakers from the Financial Services Authority of Indonesia contributed to the seminar by sharing their experiences in implementing a regulatory sandbox for financial technologies. A list of virtual seminars, participants, and their respective topics is presented in Table 3.

After the preliminary study was completed, the FGDs and in-depth interviews were divided into 3 phases: exploration, introduction, and preparation. The exploration phase determines the extent to which stakeholders recognize, are familiar with, and are receptive to the various digital technologies used to support the malaria program. In the introduction phase, the concept of a regulatory sandbox is introduced to stakeholders. In this phase, stakeholders’ acceptance of the regulatory sandbox is examined from a legal standpoint. In the preparatory phase, the research team solicited inputs from stakeholders regarding the regulatory sandbox model for e-malaria.

### Preliminary Study

During the initial phase of the study, fundamental tasks were conducted to determine subsequent phases. To facilitate future progress, we acknowledge the need to draft a memorandum of understanding with the MoH. Our team had previously received a letter of support from the Malaria Program of the MoH. Although beneficial, it was insufficient to establish a firm working relationship with the MoH. In response, Universitas Gadjah Mada and the MoH signed a memorandum of understanding. Following this, a memorandum of agreement was signed between the Faculty of Medicine, Public Health, and Nursing and the MoH’s Directorate of Vector-borne Disease and Zoonosis to ensure a comprehensive program and activities. Approximately 5 months were required to establish this legal binding, during which the team investigated numerous initiatives that could be incorporated into the development of the regulatory sandbox.

### Exploration Phase

This phase aimed to explore the opportunities and challenges associated with adopting various disruptive digital innovations to strengthen the malaria program through a regulatory sandbox. It involved discussions on potential digital technologies to support the malaria program as well as barriers to their implementation, particularly in terms of regulations. This phase took place between August 2020 and December 2020. Initially, the research team planned to conduct 3 offline FGDs; however, because of the pandemic, FGDs were eventually conducted virtually. Virtual seminars were organized as part of this phase. The summary of the FGDs conducted in this program includes an overview of the opportunities and challenges, technical considerations, and legal and social aspects, which are presented in Textbox 1.

During the exploration phase, stakeholders expressed doubts and concerns about the regulatory sandbox approach. Although not exhaustive, these concerns highlight the complexities and challenges associated with implementing a regulatory sandbox for e-malaria. Some notable quotes from stakeholders during this phase further illustrated these important reflections:

How do we ensure that the regulatory sandbox maintains the quality and safety of e-malaria innovations? It is crucial to strike a balance between promoting innovation and protecting public health.

FGD 3, health practitioner

There are legitimate concerns about data privacy and security when implementing digital health solutions. A regulatory sandbox should adequately address these issues.

FGD 6, privacy advocate

We need clear criteria for selecting healthtech startups to participate in the regulatory sandbox. This ensures that only promising and qualified innovations are tested.

FGD 1, innovation expert

These quotes signify the apprehensions and considerations raised by the stakeholders during the exploration phase. They highlighted the need for careful attention to quality assurance, data privacy, security, and a robust selection process for participation in health care start-ups. Addressing these concerns is crucial for successful implementation of regulatory sandboxes in the e-malaria domain.

### Introduction Phase

This phase marked the launch of regulatory sandbox guidelines for malaria. Following an in-depth exploration of regulatory barriers and opportunities associated with implementing disruptive digital innovations, the concept of a regulatory sandbox was introduced. The introduction phase encompassed a comprehensive discussion involving the MoH and representatives from various health care start-ups in the presence of the media. The key outcome of this phase was the official release of e-malaria regulatory sandbox guidelines that underwent an open and collaborative review process.

This phase was characterized by the culmination of various issues, including technological and legal considerations, as well as stakeholder expectations, shaping the development of regulatory sandbox guidelines (Textbox 2). Discussions and deliberations during this phase addressed a range of complex issues with the goal of producing regulatory sandbox guidelines in the health sector for the first time.

The regulatory sandbox, within the context of Indonesia as an archipelagic nation, is significant as telemedicine emerges as a supportive tool in the malaria program for enhancing external quality assurance. This is expressed in the following quote:

In island areas, telemedicine is useful. Quality assurance [for malaria microscopy] can be supported by telemedicine.

FGD 2, user of the malaria program

### Preparatory Phase

The final phase of this study involved preparation of a regulatory sandbox model for malaria. In this phase, the research team had extensive interactions with the MoH, including the malaria program, the Center for Data and Information, and the Digital Transformation Office, to prepare a team that would implement the regulatory sandbox and infrastructure required for participant registration. Input from all stakeholders was accommodated in the form of a regulatory sandbox guideline book (Textbox 3).

Throughout this study, various statements have been expressed by stakeholders reflecting the diverse perspectives and concerns held by stakeholders involved in the study. In addition to the summarized findings presented in the tables, several notable quotes highlight these perspectives. The following quotes, specifically from this phase, serve as illustrative examples:

How the Center for Data and Information MOH do start and supervise?

FGD 7, law expert

To protect the validity of e-malaria in the field, a legal base is needed. The principles of justice and humanity should form the basis of regulation.

FGD 2, user of the malaria program

Several regulations in the Ministry of Health have provided space and flexibility to adopt digital technology in supporting health programs/services. However, specific regulations and technical details have not yet been established.

FGD 5, regulator

These quotes exemplify the range of perspectives and concerns expressed by stakeholders throughout the study, highlighting the need for clear regulations, effective supervision, and the importance of aligning digital health initiatives with existing legal frameworks.

### Buy-in of the Regulator (MoH)

As a formal demonstration of buy-in, the MoH appointed a review team to oversee the e-malaria regulatory sandbox. The review team consisted of representatives from various stakeholder groups including regulatory bodies, research teams, partners, and experts in relevant fields. Their primary role was to review and assess the quality and safety of products proposed in each cluster throughout the trial. The appointment of the reviewer’s team members and delineation of their responsibilities were officially documented in decree number HK.02.02/I/3090/2021 issued by the Director General of Disease Prevention and Control of the MoH.

The MoH’s further buy-in was shown by establishing a dedicated website for the sandbox under the domain of the existing MoH website. This website serves as a centralized platform for disseminating crucial information regarding the regulatory sandbox in healthtech communities. Furthermore, the website streamlined the registration process, ensuring convenient and efficient access to interested participants to participate in the regulatory sandbox trial. The provision of this website strongly reflects the MoH’s commitment to supporting and facilitating the regulatory sandbox initiative, demonstrating its recognition of the importance of providing an accessible and user-friendly platform for stakeholder engagement.

### Key Terms of the Regulatory Sandbox

This section provides a concise explanation of the general concept of the regulatory sandbox, specifically in the context of e-malaria (Textbox 4). The key definitions of the regulatory sandbox, e-malaria, the regulatory sandbox for e-malaria, and the parties involved are provided in Textbox 4.

**Figure 2:**
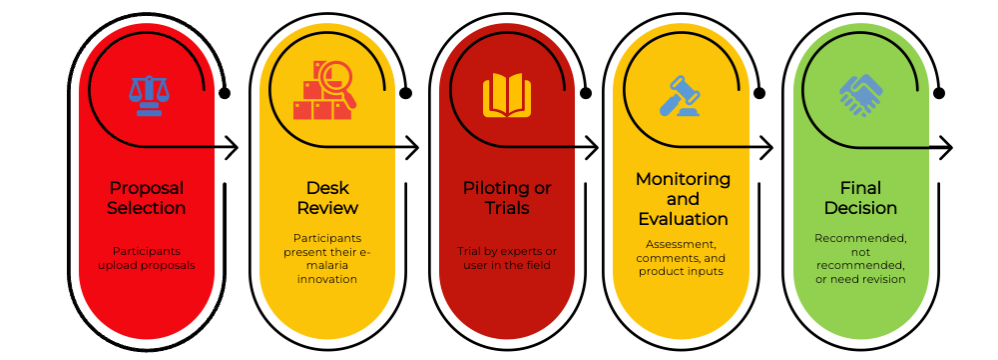
Workflow of e-malaria regulatory sandbox.

The definitions presented in Textbox 4 were derived from the e-malaria regulatory sandbox guidelines officially launched during the introductory phase. In alignment with the consensus reached among all stakeholders, the regulatory sandbox consists of 4 distinct clusters: external quality assurance, telehealth, surveillance, and supporting technologies. Numerous healthtech start-ups are expected to actively engage in successive regulatory sandbox phases, delineating distinct testing stages, as illustrated in Figure 2. This framework was designed to facilitate the advancement and assessment of disruptive technologies within the e-malaria domain, thereby guaranteeing their efficacy and adherence to the quality standards.

**Important terms.**

**Regulatory sandbox**

- A framework that enables the testing of new technologies or products in a controlled and limited-scale environment. It aims to assess the viability of a product in a real-world setting, evaluate regulatory boundaries, and gauge consumer and market responses.

**e-Malaria**

- A dedicated digital health service innovation platform designed for the malaria program. It encompasses various clusters, such as e-consultation, e-surveillance, and e-external quality assurance, which includes e-crosscheck and e-panel tests. In addition, it may include other components not mentioned in previous groups, such as e-learning.

**e-Malaria regulatory sandbox**

- A mechanism for testing the reliability, feasibility, and safety of product innovations related to malaria in a controlled environment under regulatory supervision before their public release. This approach enables policymakers to address the potential impacts and uncertainties associated with the introduction of new technologies in the field of malaria, both intentional and unintentional, on society and all elements of the ecosystem.

**Reviewers of the e-malaria regulatory sandbox**

- Individuals officially appointed by the Director General of Disease Prevention and Control, Ministry of Health (MoH), to oversee innovators or participants who registered and participated in the e-malaria regulatory sandbox trial from the initial stage until the end of the trial. The review team consisted of representatives from regulatory bodies, research teams, partner organizations, and experts in related fields. The MoH appointed 30 individuals.

**Participants of the regulatory sandbox**

- eHealth providers, innovators, and start-ups (including individuals, groups, or companies) who have registered, met the selection criteria, and actively participated in the regulatory sandbox trial.

### Stakeholders’ Perspectives: Pros and Cons

This section highlights the achievements of the research team in involving stakeholders and discusses the pros and cons of the regulatory sandbox, as expressed by different stakeholder groups. Overall, stakeholder perspectives on the proposed regulatory sandbox for e-malaria are largely positive.

Throughout the various stages of regulatory sandbox preparation, active engagement with stakeholders enables comprehensive understanding of the regulatory sandbox concept. This collaborative process resulted in the development of guidelines and establishment of a regulatory sandbox testing team. Such participatory approaches fostered stakeholder buy-in and support for integrating disruptive technologies into the malaria program despite the absence of clear legal and regulatory frameworks.

To provide a comprehensive overview of stakeholder opinions, we documented stakeholder representatives’ viewpoints on the initiative to implement an e-malaria regulatory sandbox. These perspectives, along with their respective advantages and disadvantages, are summarized in Table 4.

## Discussion

### Principal Findings

This study marks a pioneering milestone in the Indonesian health sector, with the introduction of a regulatory sandbox. In the era of digital transformation, this initiative is particularly significant, as it aligns with the MoH’s vision of implementing a regulatory sandbox for disruptive healthtech. The rapid emergence of disruptive innovations poses a profound challenge to our health care system as it empowers a broader population with limited expertise and resources to access convenient and cost-effective health care services that may harbor unknown risks [20]. By implementing a regulatory sandbox, we created a safe testing environment that allows innovators to explore and refine their technologies, whereas regulators gain invaluable insights into effectively governing these transformative advancements, as mandated by the MoH’s strategic plan [21].

However, the adoption of a regulatory sandbox encounters unique challenges in the health sector of a country that adheres to positive laws despite successful implementation in the financial sector. To ensure the successful integration of disruptive digital technologies in malaria programs, it is crucial to establish a secure space where private sector entities, including healthtech start-ups and incubators, can test their innovations under regulatory supervision [20].

Recognizing the significance of stakeholder buy-in [14], this study undertook a phased exploration to understand regulators’ perspectives and concerns regarding the regulatory sandbox approach. Our approach addresses crucial aspects such as safety, appropriateness, and potential conflicts among users, innovators, and regulators. It is worth noting that incorporating malaria, given its epidemiological change [22], into the regulatory sandbox presents its own set of challenges because the current national malaria program is solely government managed and does not involve external innovative disruptions. Therefore, the introduction of a regulatory sandbox can be viewed as a democratic endeavor aimed at creating responsive and adaptable regulations that meet the growing demand for digital health services from both the community and national programs.

According to the study by Abbot [23], regulators face 4 major challenges when dealing with disruptive technologies: the pace of scientific and technological advancements, governance of risks in the face of uncertainty, effective stakeholder engagement, and coordination within a complex regulatory system. This study has witnessed the dilemmas faced by regulators in choosing between swiftly adopting promising yet unvalidated technologies to protect populations or exercising caution and awaiting robust evidence while the contagion continues to spread [4]. Throughout our research, we fostered meaningful dialogue between regulators and innovators, recognizing that in diverse populations with varying socioeconomic, cultural, and behavioral backgrounds, this approach proves to be more effective than rigid adherence to ideology.

Our study observed the successful development of dialectical collaboration among regulators, users, and providers of digital health innovations from the exploratory phase to the preparation of the e-malaria regulatory sandbox. This collaborative effort has yielded a robust framework for regulating healthtech innovations. As such, this approach represents a more democratic step toward constructing responsive and adaptable regulations for digital health service technology. Satjipto Raharjo, a prominent law expert in Indonesia, contends that progressive law enforcement involves interpreting the underlying spirit and meaning of statutes, moving beyond literal interpretation. This approach recognizes that laws are created for the betterment of humanity and should continuously evolve to achieve justice within society. Law enforcement officials must engage in philosophical thinking to liberate knowledge, engage in theoretical discourse, and apply their insights to practice [24].

The concept of testing policy changes before enforcement aligns with sociological jurisprudence, which delves into legal studies beyond written rules to consider the practical effects and consequences of law enforcement. The elimination of malaria poses social and humanitarian challenges. Relying solely on existing positive law regulations may impede progress, as laws often lag behind current realities. Embracing a more flexible and liberal framework, such as the progressive law approach, empowers regulators to address this challenge effectively [25].

This study introduced a regulatory sandbox to facilitate contributions from external stakeholders, including innovators, civil societies, and industry professionals who are eager to contribute to malaria elimination efforts. However, these contributions would be in vain if regulators were unwilling to create a regulatory system that would nurture and embrace technological growth and innovation. Therefore, the regulatory sandbox provides an enabling environment where regulators can explore new innovations, such as digital interventions for malaria, while granting innovators the freedom to develop and advance their technologies within the boundaries of the law [26].

This study provides valuable insights into stakeholders’ perspectives on the regulatory sandbox approach in the health care sector. This adds to the existing literature on regulatory sandboxes, particularly in low- and middle-income countries, by examining the specific context of malaria. The findings reveal stakeholders’ positive expectations of this innovative regulatory approach in managing disruptive technologies. Furthermore, a comparison can be made with regulatory sandbox initiatives in other countries, such as Singapore’s Licensing Experimentation and Adaptation Programme and Malaysia’s Online Health Service Regulatory Lab [5,27]. This study opens new avenues for future research and the implementation of regulatory sandboxes in public health, offering potential solutions to the challenges of malaria elimination and other health care issues.

### Conclusions

In conclusion, the successful introduction of new innovations such as the regulatory sandbox relies heavily on the active involvement of stakeholders. Our study, which encompasses several phases of exploration, introduction, and conceptualization of the regulatory sandbox for the relatively unfamiliar domain of malaria, demonstrates the efficacy of a participatory approach. Through active engagement and collaboration with stakeholders representing diverse interests, we successfully developed the first-ever model of a regulatory sandbox for the Indonesian health sector. Future publications will delve into the challenges encountered during e-malaria regulatory sandbox trials and will provide a detailed account of the implementation process.

## Appendix

Multimedia Appendix 1 List of points discussed in the focus group discussion and in-depth interviews.

## Abbreviations

FGD: focus group discussion
MoH: Ministry of Health
WHO: World Health Organization

## Data Availability

The qualitative data supporting the findings of this study are available from the corresponding author upon reasonable request. Some data may not be available owing to privacy or ethical restrictions.
