# Supplementary material for: Introducing a Regulatory Sandbox Into the Indonesian Health System Using e-Malaria as a Use Case: Participatory Action Study
Source: J Med Internet Res. 2023 Dec 5;25:e47706. doi: 10.2196/47706 (PMC10731549; doi:10.2196/47706)
Supplement: Multimedia Appendix 1 [file jmir_v25i1e47706_app1.docx]

| **Points for discussion and in-depth interview** | **FGD** |
| --- | --- |
| How is the community's readiness to use digital technology in the health sector so far to reduce malaria cases? See experience of health digital technology in Indonesia | 1 |
| What do you think if the process of diagnosing, cross-checking, and testing the malaria panel is conducted digitally? | 2 |
| Discussion on the legal aspects of personal data protection in e-health, the application of confidentiality and prudence principles in the regulatory sandbox | 3,5 |
| Discussion on e-health technology and regulatory sandbox from the perspective of values, norm, and legal views | 3,5 |
| What is the experience in providing e-health (telemedicine) services during the Covid-19 pandemic | 4 |
| Have you ever been asked to register startup compliance at the Ministry of Communication and Information by the association? If you have registered, what was the process like? Do you think the registration process is important? Why? | 4 |
| Discussion on risk mitigation in the e-health sector and the regulatory sandbox | 5 |
| What form of regulation will be used as the legal basis and implementation of e-malaria governance through the Regulatory Sandbox | 6 |
| Discussion on the clauses for managing e-malaria risk mitigation in the Regulatory Sandbox study compiled and Ministry of Health Regulation draft | 6 |
| What is the scope and direction of the outreach of academic papers and the draft of the minister of health in the study of e-malaria governance through the Regulatory Sandbox that has been prepared | 7 |
